# YEATS4 promotes the tumorigenesis of pancreatic cancer by activating beta-catenin/TCF signaling

**DOI:** 10.18632/oncotarget.15633

**Published:** 2017-02-23

**Authors:** Chen Jixiang, Dang Shengchun, Qu Jianguo, Mao Zhengfa, Fan Xin, Wang Xuqing, Zhang Jianxin, Cui Lei

**Affiliations:** ^1^ General Surgery Department, Affiliated Hospital, Jiangsu University, Zhenjiang City, Jiangsu Province, 212000 China

**Keywords:** YEATS4, pancreatic cancer, beta-catenin/TCF signaling, cancer metastasis

## Abstract

Beta-catenin/TCF signaling has been reported to promote the growth and metastasis of pancreatic cancer cells. However, the regulation for the beta-catenin/TCF transcriptional complex remains largely unknown. Here, we have found that YEATS4 is a positive regulator for Beta-catenin/TCF signaling. The expression of YEATS4 was elevated in clinical pancreatic cancer samples and pancreatic cancer mouse model. Up-regulation of YEATS4 promoted the growth, migration and invasion of pancreatic cancer cells, while knocking down the expression of YEATS4 inhibited the growth, migration, invasion and metastasis of pancreatic cancer cells. Moreover, the mechanism study revealed that YEATS4 interacted with beta-catenin and activated beta-catenin/TCF signaling. Furthermore, knocking down the expression of YEATS4 impaired the malignant transformation of normal pancreatic cells (HPDE6C7) by the oncogenic Ras. Taken together, our study demonstrated the oncogenic roles of YEATS4 in the progression of pancreatic cancer by activating beta-catenin/TCF signaling and suggested that YEATS4 might be a promising therapeutic target for pancreatic cancer.

## INTRODUCTION

Numerous studies have demonstrated the roles of Wnt/beta-catenin signaling in cell proliferation, differentiation and motility [1]. Beta-catenin is the key molecule in the Wnt/beta-catenin pathway [2]. In the resting state, the protein level of beta-catenin is tightly controlled by the destruction complex which contained APC, Axin and GSK3beta. The stimulation of Wnt ligand releases beta-catenin from the destruction complex, which leads to the cytoplasmic accumulation and nuclear localization of beta-catenin. In the nucleus, beta-catenin formes a complex with TCF4 (T-cell factor 4) and promotes the transcription of multiple genes [3]. Up to date, the regulation for beta-catenin/TCF complex remains largely unknown.

Aberrant activation of Wnt/beta-catenin signaling has been observed in various cancer types [4–6]. Nuclear localized beta-catenin has been found in about 60% clinical pancreatic cancer samples, suggesting the over-activation of beta-catenin/TCF signaling in the progression of pancreatic cancer [7]. Moreover, lots of studies have shown that activation of beta-catenin/TCF signaling promoted the growth, migration and metastasis of pancreatic cancer cells [8–10]. In the recent years, a few of regulators for the beta-catenin destruction complex have been identified, such as Mucin, ATDC and so on, which help us better understand the regulation of beta-catenin destruction complex [11, 12]. However, how the nuclear beta-catenin/TCF complex was regulated remains poorly understood.

YEATS4 was initially found in the nucleoli [13]. It has high sequence homology to human MLLT1 and MLLT3 proteins, indicating that the encoded protein might represent a transcription factor [14]. This protein is thought to be required for RNA transcription by interacting with the general transcriptional factor TFIIF [15]. Recently, YEATS4 has been shown to be involved in the tumorigenesis. YEATS4 is a novel oncogene amplified in non-small cell lung cancer that inhibits the p53 pathway [16]. Furthermore, the expression of YEATS4 has been reported to lead to drug resistance of ovarian cancer cells [17]. Also, knockdown of YEATS4 inhibited colorectal cancer cell proliferation and induces apoptosis [18]. The functions of YEATS4 in pancreatic cancer are unknown.

In this study, we have examined the expression pattern of YEATS4 in pancreatic cancer, investigated the functions of YEATS4 and elucidated the underlying molecular mechanism.

## RESULTS

### Up-regulation of YEATS4 was found in both human pancreatic cancer and pancreatic cancer mouse model

**Figure 1:**
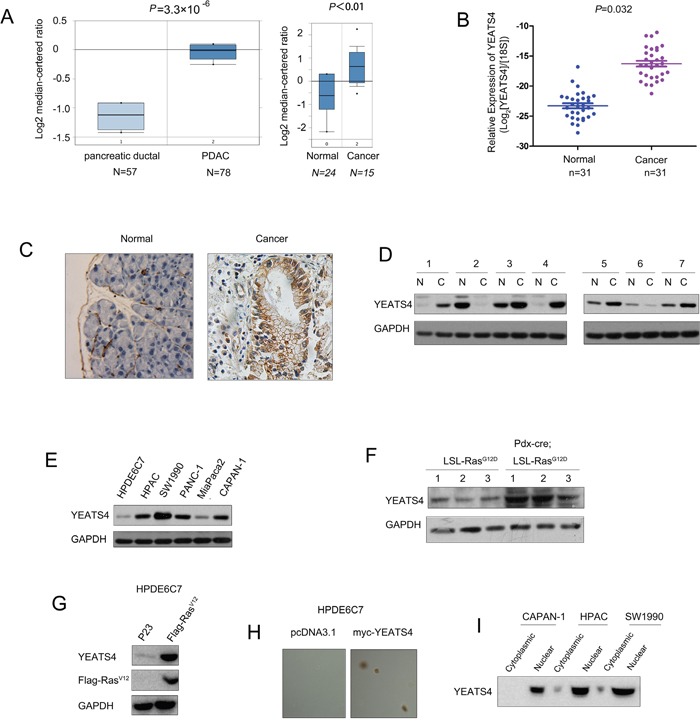
The expression of YEATS4 was elevated in pancreatic cancer **A**. The up-regulation of YEATS4 in pancreatic adenocarcinoma was found in two Oncomine database. Boxplot of YEATS4 mRNA Log2 expression levels was evaluated in Oncomine database. One study contained 57 normal pancreatic tissues, 78 pancreatic cancer tissues. Another study contained 24 normal pancreatic tissues, 15 pancreatic cancer tissues. **B**. The mRNA level of YEATS4 wasincreased in pancreatic cancer tissues compared with the paired normal tissues. The YEATS4 mRNA level was examined in 31 pancreatic cancer tissues and the paired normal tissues using Real-time PCR. The expression of YEATS4 mRNA level was normalized to 18S. **C**. The expression of YEATS4 protein in pancreatic cancer tissues and paired non-cancerous tissues was examined using immunohistochemistry. **D**. The expression of YEATS4 protein in 7 pancreatic cancer tissues and paired non-cancerous tissues was examined using western blot analysis. **E**. The expression of YEATS4 in normal pancreatic cells (HPDE6C7) and pancreatic cancer cells (HPAC, SW1990, PANC-1, miapaca2 and Capan-1). **F**. The expression of YEATS4 protein was up-regulated in the pancreatic tissues of the mouse Pdx-Cre; Ras^G12D^. Three mice for each group were used in this study. *, *P*< 0.05; **, *P*<0.01. **G**. The expression of YEATS4 in normal pancreatic cells (HPDE6C7) was induced by overexpression of Ras^V12^. **H**. The expression of YEATS4 promoted the growth of normal pancreatic cells (HPDE6C7) in soft agar. **I**. The expression of YEATS4 was detected in the nucleus extract of SW1990, HPAC and Capan-1.

To study the expression profile of YEATS4 in the pancreatic cancer, we first turned to the Oncomine Database. Two independent studies have shown the up-regulation of YEATS4 in the pancreatic cancer (Figure 1A). We further confirmed the up-regulation of YEATS4 by examining the mRNA level of YEATS4 in 31 pancreatic cancer clinical samples and 31 adjacent non-cancerous tissues. Obviously, the mRNA level of YEATS4 was significantly higher in cancer tissues when compared with the paired non-cancerous tissues (Figure 1B). Next, we examined the protein level of YEATS4 in human pancreatic cancer samples by immunohistochemistry staining and western blot analysis (Figure 1C and 1D). The accumulation of YEATS4 in the cytoplasm and nucleus of pancreatic cancer cells was found (Figure 1C). Furthermore, elevated YEATS4 protein level was observed in about 70% (5/7) pancreatic cancer samples in the western blot analysis (Figure 1D). Additionally, we examined the YEATS4 protein level in normal pancreatic cells and a panel of pancreatic cancer cell lines. Comparing with the normal pancreatic cells (HPDE6C7), up-regulation of YEATS4 in pancreatic cancer cells (HPAC, SW1990, MiaPaca2, PANC-1 and Capan-1) was observed (Figure 1E). These findings suggested the up-regulation of YEATS4 in human pancreatic cancer. Moreover, we examined the protein level of YEATS4 in the pancreatic cancer mouse model (Pdx-Cre; L-S-L-Ras^G12D^), which was driven by the oncogenic Ras^G12D^. The crossing with Pdx-cre deleted the stop condon upstream of Ras^G12D^ coding sequence and induced pancreatic intraepithelial neoplasia (PanIN) 8 months after birth. Clearly, the protein level of YEATS4 was increased in the PanIn tissues (Figure 1F).

In addition, we examined whether Ras signaling induced the expression of YEATS4. As shown in Figure 1G, the active Ras signaling promoted the expression of YEATS4. Furthermore, over-expression of YEATS4 transformed the normal pancreatic cells HPDE6C7 (Figure 1H). Moreover, it has been found that YEATS4 was mainly expressed in the nucleus (Figure 1I). Taken ogether, these data suggested the up-regulation of YEATS4 in the pancreatic cancer.

### YEATS4 promoted the growth, migration, colony formation and invasion of pancreatic cancer cells

**Figure 2:**
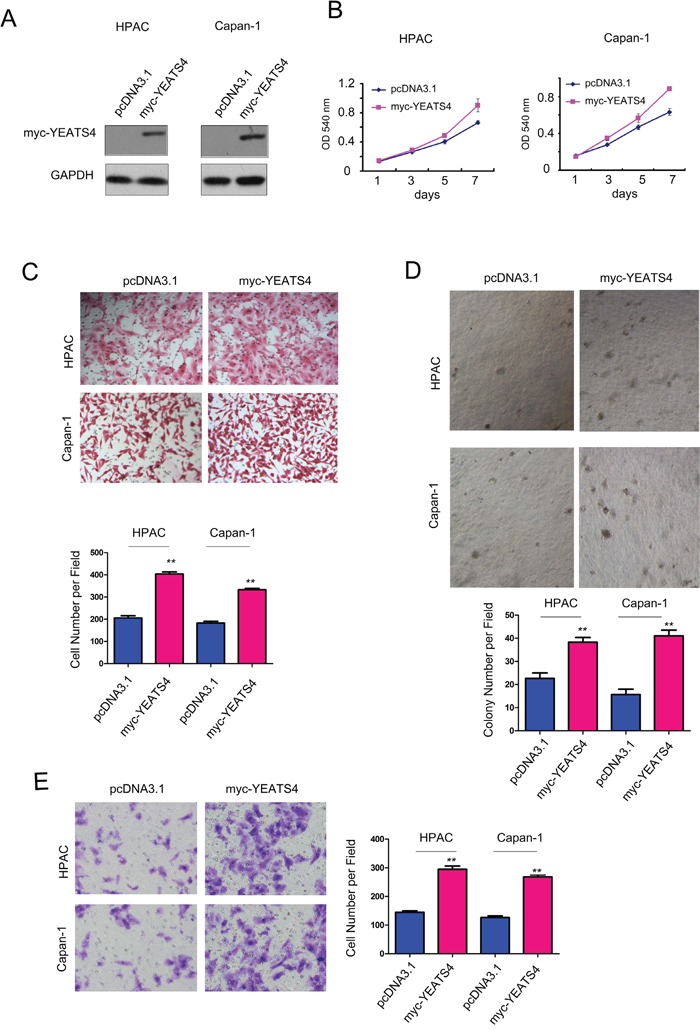
YEATS4 promoted the growth, migration and invasion of pancreatic cancer cells **A**. Forced expression of YEATS4 in HPAC and Capan-1 cells. **B**. The effects of YEATS4 on the growth of HPAC and Capan-1 cells in the MTT assay. **C**. The effects of YEATS4 on the migration of HPAC and Capan-1 cells in the Boyden chamber assay. **D**. The effects of YEATS4 on the anchorage-independent growth of HPAC and Capan-1 cells in the soft agar assay. **E**. The effects of YEATS4 on the invasion of HPAC and Capan-1 cells.

**Figure 3:**
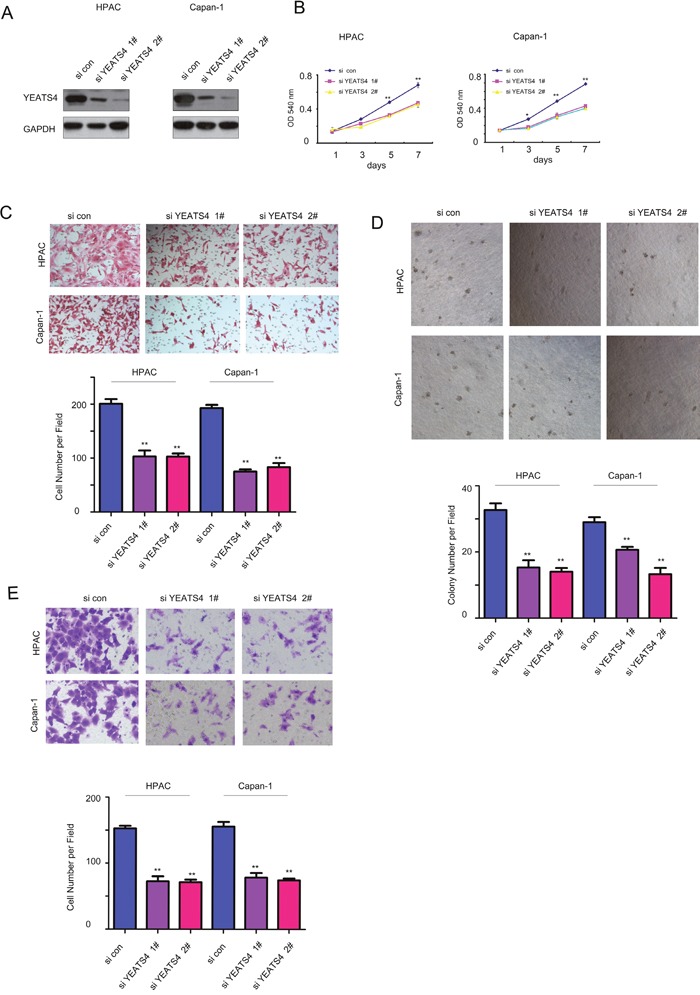
Knocking down the expression of YEATS4 inhibited the growth, migration and invasion of pancreatic cancer cells **A**. YEATS4 was knocked down in HPAC and Capan-1 cells. **B**. The effects of down-regulating YEATS4 on the growth of HPAC and Capan-1 cells in the MTT assay. **C**. The effects of down-regulating YEATS4 on the migration of HPAC and Capan-1 cells in the Boyden chamber assay. **D**. The effects of down-regulating YEATS4 on the anchorage-independent growth of HPAC and Capan-1 cells in the soft agar assay. **E**. The effects of down-regulating YEATS4 on the invasion of HPAC and Capan-1 cells.

To study the roles of YEATS4 in the tumorigenesis of pancreatic cancer, we first over-expressed YEATS4 in HPAC and Capan-1 cells and confirmed the exogeneous expression of YEATS4 (myc-YEATS4) by western blot analysis (Figure 2A). The effects of YEATS4 on the growth, migration, colony formation and invasion of pancreatic cancer cells were evaluated by MTT assay, Boyden chamber and soft agar assay, respectively (Figure 2B–2E). Obviously, forced expression of YEATS4 in HPAC and Capan-1 cells promoted cell growth (Figure 2B). Moreover, overexpression of YEATS4 in HPAC and Capan-1 cells improved the motility of cancer cells demonstrated by the cell migration assay and invasion assay (Figure 2C and 2E). Furthermore, YEATS4 promoted the anchorage-independent growth of HPAC and Capan-1 cells in the soft agar (Figure 2D). These data suggested that YEATS4 promoted the tumorigenesis of pancreatic cancer. To better understanding the roles of YEATS4 in pancreatic cancer, we knocked down its expression in HPAC and Capan-1 cells using two independent si RNA sequence (Figure 3A). Knocking down the expression of YEATS4 in HPAC and Capan-1 cells inhibited the growth of cancer cells in liquid culture (Figure 3B), as well as the migration and invasion of pancreatic cancer cells based on the Boyden chamber assay (Figure 3C and 3E). Most importantly, down-regulation of YEATS4 impaired the anchorage-independent growth of HPAC and Capan-1 cells (Figure 3D). Collectively, these results demonstrated that YEATS4 promoted the growth, migration, colony formation and invasion of pancreatic cancer cells.

### YEATS4 promoted the migration, colony formation and invasion of pancreatic cancer cells by activating beta-catenin/TCF signaling

**Figure 4:**
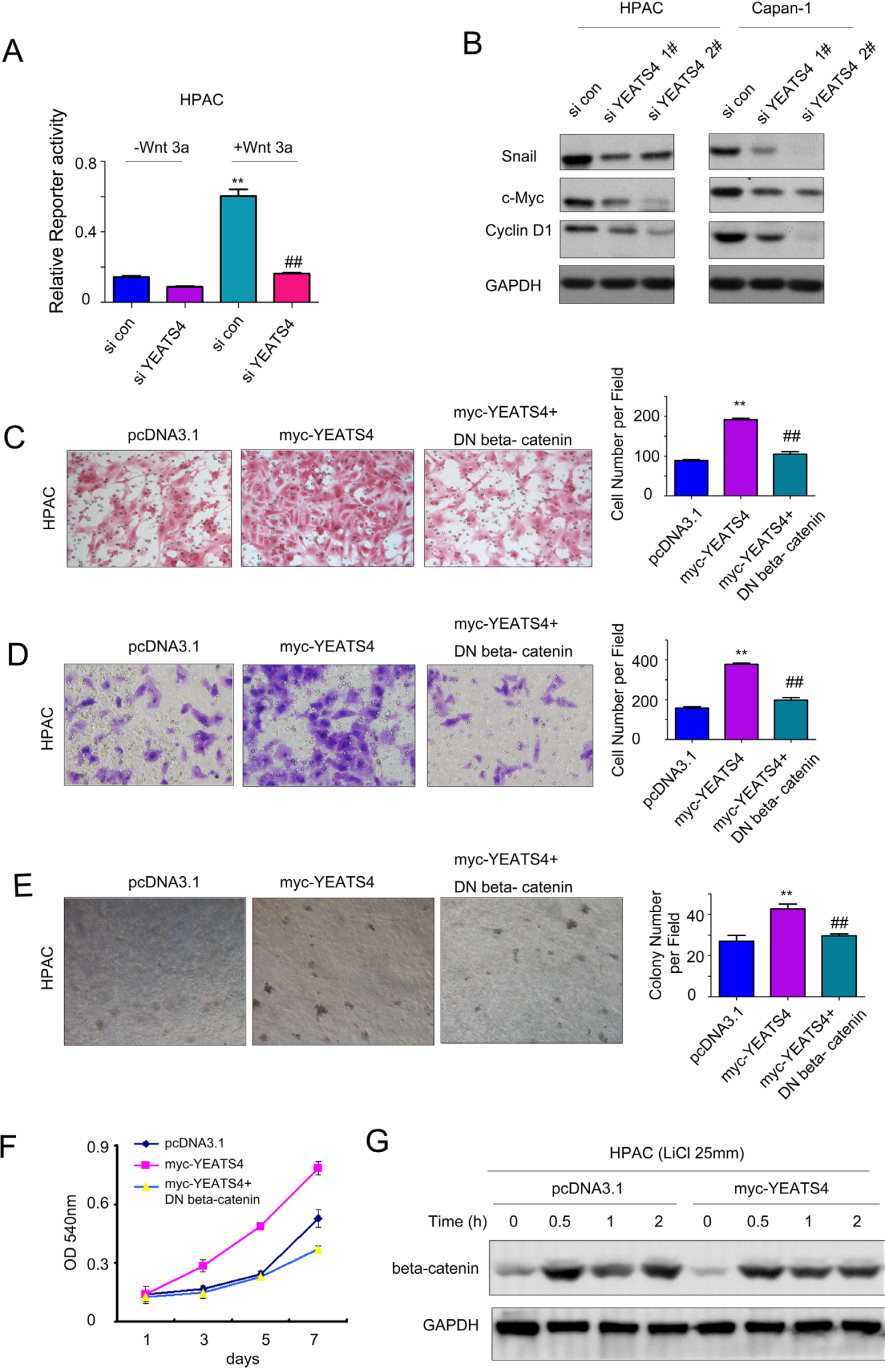
YEATS4 activated beta-catenin/TCF signaling in pancreatic cancer cells **A**. Knocking down the expression of YEATS4 inhibited the activity of Topflash, an indicator for beta-catenin/TCF signaling, both at the basal level and upon the stimulation of wnt3a. **B**. Knocking down the expression of YEATS4 down-regulated the expression of Cyclin D1, Snail and c-Myc. **C**. Dominant negative beta-catenin, the negative regulator for beta-catenin/TCF signaling, rescued the effects of YEATS4 on the migration of HPAC cells. **D**. Dominant negative beta-catenin rescued the effects of YEATS4 on the invasion of HPAC cells. **E**. Dominant negative beta-catenin rescued the effects of YEATS4 on the anchorage-independent growth of HPAC cells. **F**. Dominant negative beta-catenin rescued the effects of YEATS4 on the growth of HPAC cells in liquid culture. **G**. YEATS4 did not elevate the protein level of beta-catenin. HPAC cells were treated with LiCl for different time and the beta-catenin protein level was examined.

To explore the underlying mechanisms for the oncogenic roles of YEATS4 in the pancreatic cancer, we screened the effects of YEATS4 on the activity of several pathways using lusiferase reporter assay. Down-regulation of YEATS4 impaired the activity of Topflash reporter (an indicator for beta-catenin/TCF signaling) both at the basal level and upon the stimulation of wnt3a (Figure 4A). Moreover, knocking down the expression of YEATS4 in HPAC and Capan-1 cells decreased the expression of several target genes downstream beta-catenin/TCF signaling, including Snail, c-myc and cyclin D1 (Figure 4B). To examine whether YEATS4 promoted the migration, invasion and anchorage-independent growth of pancreatic cancer cells through activating beta-catenin/TCF signaling, we investigated whether dominant negative beta-catenin (DN beta-catenin) could rescued the biological functions of YEATS4. Over-expression of DN beta-catenin abolished the promoting effects of YEATS4 on the migration, invasion and growth of HPAC cells (Figure 4C-4F). In addition, we test whether the expression of YEATS4 elevated the protein level of beta-catenin. As shown in Figure 4G, over-expression of YEATS4 did not elevate the beta-catenin protein level upon the treatment of LiCl. In summary, these data suggested that YEATS4 promoted the migration, colony formation and invasion of pancreatic cancer cells by activating beta-catenin/TCF signaling.

### YEATS4 interacted with beta-catenin in pancreatic cancer cells

**Figure 5:**
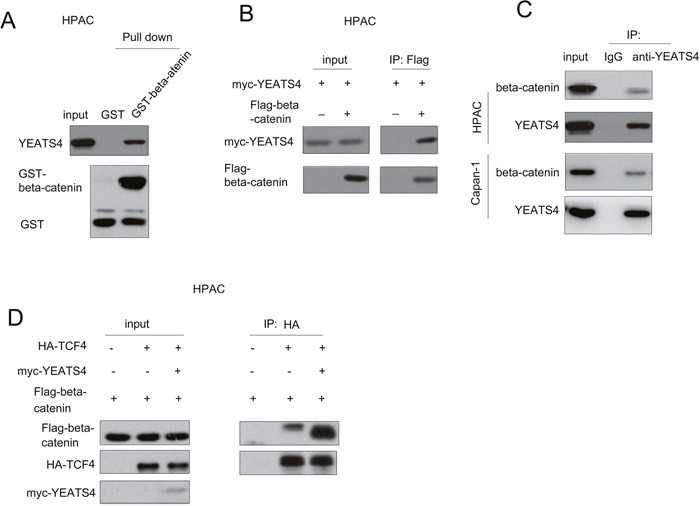
YEATS4 interacted with beta-catenin **A**. GST pull-down assay using HPAC cell lysate demonstrated the interaction between GST-beta-catenin and YEATS4. **B**. Exogenously expressed beta-catenin (Flag-beta-catenin) and YEATS4 (myc-YEATS4) formed a complex in HPAC cells in the immunoprecipitation assay. **C**. Endogenously expressed beta-catenin and YEATS4 formed a complex in HPAC cells and Capan-1 cells. **D**. YEATS4 enhance the interaction between beta-catenin and TCF4 in the immunoprecipitation assay.

Next, we studied how YEATS4 activated the beta-catenin/TCF signaling. Considering the nuclear localization of YEATS4 in the pancreatic cancer, we first tested the interaction between YEATS4 and beta-catenin/TCF4 transcriptional machinery. As shown in Figure 5A, the fusion protein GST-beta-catenin interacted with endogenously expressed YEATS4 in HPAC cells, indicating the interaction between YEATS4 and beta-catenin (Figure 5A). In addition, the ectopically expressed YEATS4 (myc-YEATS4) and beta-catenin (Flag-beta-catenin) formed a complex in HPAC cells (Figure 5B). Most importantly, endogenously expressed YEATS4 and beta-catenin interacted with each other (Figure 5C).

Upon the stimulation of wnt3a, beta-catenin and TCF4 formed a complex to activate the expression of downstream genes. We next examined whether he expression of YEATS4 enhanced the interaction between TCF4 and beta-catenin. Forced expression of YEATS4 enhanced the interaction between TCF4 and beta-catenin (Figure 5D). Collectively, these data demonstrated that YEATS4 activated beta-catenin/TCF signaling by enhancing the interaction between beta-catenin and TCF4.

### YEATS4 promoted the metastasis of pancreatic cancer cells *in vivo*

**Figure 6:**
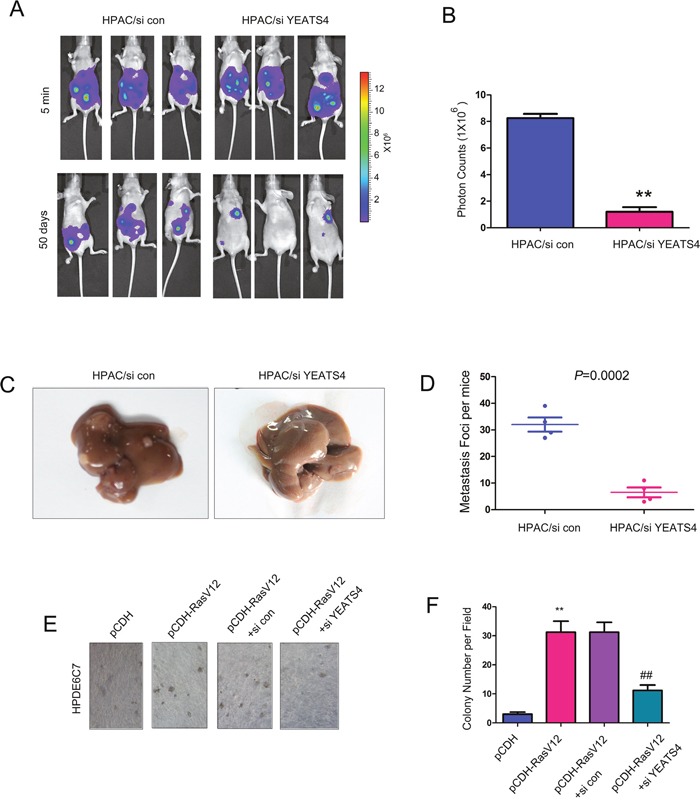
Down-regulating YEATS4 inhibited the metastasis of pancreatic cancer cells **A-B**. Knocking down the expression of YEATS4 inhibited the metastasis of HPAC cells. Cells were injected into the nude mice through the left ventricle of the heart. 50 days later, the intensity of the fluorescence was quantified. **C-D**. Knocking down the expression of YEATS4 inhibited the distant seeding and metastaic foci formation of HPAC cells in the liver tissues. **E-F**. Knocking down the expression of YEATS4 inhibited the malignant transformation of normal pancreatic cells HPDE6C7 driven by Ras^V12^. HPDE6C7 was forced expression of Ras^V12^ and then knocked down the expression of YEATS4. The soft agar assay was performed.

In the next study, we examined the effects of YEATS4 on the metastasis of pancreatic cells. HPAC cells were forced to express a luciferase gene, which enabled the tracing of HPAC cells *in vivo* after administration of luciferin (the substrate of luciferase). The HPAC cells knocking down the expression of YEATS4 (HPAC/si YEATS4) and the control cells (HPAC/si con) were injected into the left ventricle of the nude mice and the metastatic foci were monitored. 50 days after the injection, more metastatic foci were observed in the mice injected with the control cells (Figure 6A and 6B). The metastasis to the liver is one of the features of pancreatic cancer cells. It was found that knocking down the expression of YEATS4 impaired the metastasis of HPAC cells to the liver (Figure 6C and 6D). Moreover, the up-regulation of YEATS4 in the pancreatic cancer mouse model (Figure 1F) prompted us to investigate whether YEATS4 was essential for the transformation of HPDE6C7 driven by Ras^G12D^. As shown in Figure 6E, knocking down the expression of YEATS4 impaired anchorage-independent growth of HPDE6C7 induced by Ras^V12^ (Figure 6E). Taken together, these observations indicated that YEATS4 promoted the metastasis of pancreatic cancer cells and was necessary for the malignant transformation of normal pancreatic cells.

## DISCUSSION

In this study, we have demonstrated that YEATS4 promoted the growth, migration and invasion of pancreatic cancer cells by activating beta-catenin/TCF signaling. The expression of YEATS4 was increased in the clinical pancreatic cancer samples, which was confirmed by the oncomine database and our experimental results, suggesting that YEATS4 might be diagnosis marker for PDAC. Although previous studies have reported the functions of YEATS4 in the cell growth and apoptosis [18], the roles of YEATS4 in the migration, invasion and metastasis of cancer cells remain unknown. Our study demonstrated the effects of YEATS4 on the motility of cancer cells, suggesting the regulation of cell motility by YEATS4. In this study, knocking down YEATS4 has shown to down-regulate the expression of Snail, a master of EMT (epithelial-mesenchymal transition), suggesting that YEATS4 might promote the motility of pancreatic cancer cells by inducing EMT.

Another important finding of this study is identifying YEATS4 as a bingding partner of beta-catenin. The replacement of Grouch with beta-catenin for the binding of TCF4 activated the transcription of multiple genes [19]. In this study, we have found that YEATS4 enhanced the interaction between beta-catenin and TCF4, suggesting that YEATS4 might compete with Grouch for the binding of beta-catenin. It has been reported that YEATS4 negatively regulated the P53-P21 pathway in the lung cancer [16]. Combining with our study, YEATS4 might be a hub for the transduction of various pathways. Therefore, inhibiting the activity of YEATS4 might be a promising strategy for the cancer therapy.

Oncogenic mutation of Ras on the 12^th^ amino acid occurred in most of the pancreatic cancer samples [20]. Directly targeting oncogenic Ras is unsuccessful up to date. In the present study, YEATS4 was up-regulated in the pancreatic cancer mouse model, knocking down the expression of YEATS4 impaired the malignant transformation of normal pancreatic cells HPDE6C7, and YEATS4 is a target and effector of oncogenic Ras signaling. Therefore, YEATS4 might be a promising therapeutic target for pancreatic cancer.

In summary, our study demonstrated that YEATS4 promoted the progression of pancreatic cancer by activating beta-catenin/TCF signaling. Although our data are very indicative, the further study using YEATS4 knocking out mice would provide novel insights into its functions.

## MATERIALS AND METHODS

### Pancreatic cancer samples

31 pancreatic cancer samples and paired normal tissues were obtained from patients who subjected surgery at Jiangsu University affiliated hospital and stored at -80°C. The consent forms were got from the patients. This study was approved by the ethics committee of the hospital.

### Cell culture

Pancreatic cancer cell lines (HPAC, Capan-1, MIAPaca2, SW1990 and PANC-1) and normal human pancreatic cell line (HPDE6C7) were obtained from ATCC (American Typical Culture Center). All cells were cultured in DMEM medium supplemented with 10% fetal bovine serum (GIBCO), 100units/mL penicillin and 100μg/mL streptomycin in an incubator with 5% CO_2_ at 37°C.

### Q-PCR

Total RNA was exacted from the clinical tissues using TRIzol. The reverse transcription kit was used to prepare the complementary DNA (cDNA). The expression of YEATS4 in the pancreatic cancer tissues and paired normal tissues was examined by quantitative real-time PCR using SYBR® Green Realtime PCR Master Mix (TOYOBO) following the instructions of the manufacturer. Sequences of quantitative real-time PCR primers are listed as follows: 18S Forward primer: 5′-TAAATCAGTTATGGTTCCTT -3′; 18S Reverse primer: 5′-CGACTACCATCGAAAGTTGA-3′; YEATS4 Forward primer: 5′-AGAGAATG GCCGAATTTGGG-3′; YEATS4 Reverse primer: 5′-TCATAGAACTCTGAAACCAC-3′

### Immunohistochemistry

Xylene and ethanol was used to deparaffinized and rehydrated the paraffin-embedded tissue. Endogenous peroxidase activity was blocked with 0.35% H2O2 solution. Antigens retrieve was performed using microwaving. Non-specific binding was blocked by 1% BSA solution Sections were stained with YEATS4 antibody and visualized with secondary antibody (Envision, Gene Techenology). Slides were then developed with DAB andcounterstained with hematoxylin.

### Immunoblotting

The cellular proteins were resolved by SDS-PAGE after lysed by the RIPA buffer. The proteins were transferred to the PDVF membrane. After blocking with the 3% BSA solution, the membrane was incubated with following primary antibodies over night: anti-YEATS4 (Abcam), anti-Snail (Cell Signaling Technology), anti-Cyclin D1 and anti-c-Myc (Cell Signaling Technology), anti-GAPDH (Santa Cruz). The membranes were washed with TBST solution and incubated with the secondary antibody for 1 hour at the room temperature. The protein was visualized by ECL kit.

### Pull-down assay

The coding sequence of beta-catenin was cloned into the expression vector pGEX-4T-1. The fusion protein GST-beta-catenin was purified. The whole cell lysates of HPAC were prepared in 50 mM Tris-Cl (pH 7.5), 150 mM NaCl, 0.1% NP40 and protease inhibitor cocktail. 5 μg GST-beta-catenin fusion protein and 500 μg cell lysates were incubated at 4°C over night. 50 μl of glutathione-Sepharose-4B beads were added to the samples and incubated at 4°C for 1 hr to capture the GST fusion proteins. After washing with lysis buffer three times, the proteins were eluted in Laemmli buffer and analyzed by SDS-PAGE.

### Reporter assay

HPAC Cells were grown to a subconfluent density. 16 hours later, the reporter assays were performed using 0.1 μg of Topflash, 0.5 μg of expression vector, and 0.05 μg of TK Renilla luciferase (internal control for transfection efficiency). 48 hours later, cells were treated with Wnt3a protein for 8 hours. Then, cell lysates were prepared and the reporter activity was measured using the dual-luciferase reporter assay system (Promega).

### Plasmids

The coding sequence of YEATS4 was amplified by PCR and inserted into the expression vector pcDNA3.1 to obtain the myc tagged YEATS4. The coding sequence of beta-catenin was amplified by PCR and inserted into the expression vector pCMVTag2B to obtain the Flag tagged beta-catenin. The coding sequence of TCF4 was amplified by PCR and inserted into the expression vector pCMV-HA to obtain the HA tagged TCF4.

### YEATS4 si RNA

RNAi lenti-virus particles (sicon and si YEATS4) were purchased from GeneChem (China). Cells were infected with the indicated lenti-virus particles of the same MOI for 24 hours and then stable knock-down cells were selected with the medium containing puromycin for at least a week.

### MTT assay

10^5^ cells/well were plated in 96-well plates. Cell growth was determined using the 3-(4,5-methylthiazol-2-yl)-2,5-diphenyltetrazolium bromide (MTT) colorimetric growth assay for a week. Every other day, cell growth was determined by adding MTT solution (50μg/well) for 4h. Cellular MTT was resolved with DMSO and was measured at 540 nm. All experiments were performed in triplicates.

### Boyden chamber assay

Boyden chamber was used to evaluated the motility of pancreatic cells. Cells (2×10^5^) suspended in 0.05ml medium containing 1% FBS were placed in the upper chamber, and the lower chamber was loaded with 0.152ml medium containing 10% FBS acting as the chemoattractant. 12 hours later, cells migrated to the lower surface of filters was detected with traditional hematoxylin and eosin (H&E) staining. The experiments were repeated for three times. Five random visual fields were counted for each sample and the average was determined.

### Soft agar assay

In soft agar assay, 5000 cells/well were suspended in the upper layer (0.35% agarose and 10% FBS in DMEM) in 6-well plates. The plates were coated with bottom layer (0.5% agarose and 10% FBS in DMEM). After 14 days of incubation, the colonies were counted and measured. All of the experiments were done at least three times.

### Mice model

Mice were housed and treated after being approved by the Institutional Animal Care and Use Committee of Jiangsu University. *Kras^G12D^* and *Pdx-Cre* mice were obtained from Jackson Lab (Koch Institute for Integrative Cancer Research, Cambridge, MA). Pancreatic cancer mouse models *Pdx-Cre; Kras^G12D^* mice were generated by crossing *Kras^G12D^* and *Pdx-Cre* mice.

### Statistical analysis

Statistical analysis was performed by the Student *t*-test (two-tailed) using Prism GraphPad software. Differences with *P* < 0.05 were considered statistically significant. Data were represented as mean±SEM.

## Abbreviations

PDAC: Pancreatic ductal adenocarcinoma
TCF4: T-cell factor 4
ATCC: American Type Culture Collection
DMEM: Dulbecco's modified Eagle's medium
GFP: green fluorescence protein
FBS: fetal bovine serum
RT-PCR: Reverse transcription quantitive PCR
cDNA: Complementary DNA
PBS: phosphate-buffered saline
